# Application of HER2 peptide vaccines in patients with breast cancer: a systematic review and meta-analysis

**DOI:** 10.1186/s12935-021-02187-1

**Published:** 2021-09-15

**Authors:** Zicong You, Weijun Zhou, Junyan Weng, Haizhan Feng, Peiqiao Liang, Yuhua Li, Fujun Shi

**Affiliations:** 1 Department of Breast Surgery, Zhujiang Hospital, Southern Medical University, No. 253, Industrial Avenue, Haizhu District, Guangzhou, 510282 China; 2 Department of Thoracic and Breast Surgery, Foshan Hospital of Traditional Chinese Medicine, Guangzhou University of Chinese Medicine, No.6,Qinren Road,Chancheng District, Foshan, 528000 China; 3 Department of Hematology, Zhujiang Hospital, Southern Medical University, No.253, Industrial Avenue, Haizhu District, Guangzhou, 510282 China

**Keywords:** HER2, Breast cancer, Vaccine, Systematic review, Meta-analysis

## Abstract

**Background:**

The E75 and GP2 vaccines are the few therapeutic vaccines targeting HER2 currently under clinical research for patients with breast cancer.

**Methods:**

Databases, including the Cochrane Library, PubMed, Medline, Embase, and Web of Science, were used to retrieve clinical studies on E75 and GP2 vaccines. Retrieval time was from the beginning of database construction until May 31st, 2021.

**Results:**

A total of 24 clinical studies were included in this analysis, including 1704 patients in the vaccinated group and 1248 patients in the control group. For the E75 vaccine, there were significant differences between the vaccinated group and the control group in the delayed-type hypersensitivity reaction (SMD = 0.685 95% CI 0.52–0.85, P_Heterogeneity_ = 0.186, P_DTH_ < 0.05) and the change in CD8^+^ T-cell numbers (SMD = − 0.864, 95% CI − 1.02 to − 0.709, P_Heterogeneity_ = 0.085, P_CD8+ T cell_ < 0.05) before and after injection. For the GP2 vaccine, there was a significant difference between the vaccinated group and the control group in the change in CD8^+^ T-cell numbers (SMD = − 0.584, 95% CI − 0.803 to − 0.294, P_Heterogeneity_ = 0.397, P_CD8+ T cell_ < 0.05) before and after injection. In addition, the clinical outcomes, including recurrence rate (RR = 0.568, 95% CI 0.444–0.727, P_Heterogeneity_ = 0.955, P_Recurrence_ < 0.05) and disease-free survival rate (RR = 1.149, 95% CI 1.050–1.256, P_Heterogeneity_ = 0.003, P_DFS_ < 0.05), of the E75-vaccinated group were different from those of the control group. However, we found that the overall survival rate with the E75 vaccine (RR = 1.032, 95% CI 0.998–1.067, P_Heterogeneity_ = 0.476, P_OS_ > 0.05) was not different between the two groups. Local and systemic toxicity assessments of the two vaccines showed minimal side effects.

**Conclusions:**

The E75 vaccine was effective and safe in patients with breast cancer. The GP2 vaccine could elicit a strong immune response, but more trials are needed to confirm its clinical efficacy.

## Introduction

Breast cancer is a malignant tumor originating in the ductal epithelium of the breast. In the United States, the estimated number of new cases of breast cancer in 2020 was 276,480, accounting for approximately 30.28% of all cases of primary tumors in women [1]. In recent years, great progress has been made with peptide vaccines against tumors, which may provide a potential treatment for patients with breast cancer [2]. The mechanism of peptide vaccines mainly contains three parts. First, antigen-presenting cells (APCs) ingest the peptide after injection. Second, CD8^+^ T-cells recognize APCs and generate specific cytotoxic lymphocytes (CTLs). Third, CTLs specifically recognize tumor cells expressing antigen and then release perforin and cytokines to dissolve the tumor cells [3]. Human epidermal growth factor receptor-2 (HER2) is an important regulator of the growth and development of HER2-positive breast cancer cells and is mainly expressed in embryos. Only a small amount of HER2 has been detected in normal breast cells [4]. However, approximately 20–30% of patients with breast cancer overexpress HER2 [5], which make it a popular target for the design of tumor immunotherapy.

None of the therapeutic vaccines have been formally applied in breast cancer clinical treatment, but clinical trials have been actively conducted with vaccines having different mechanisms and effects [6]. Among them, vaccines targeting HER2 have been further studied in multiple trials. HER2 peptide vaccines mainly include E75 (p369–377), GP2 (p654–662), and AE37 (p776–790). E75 is p369 with KIFGSLAFL amino acid sequence. The E75 vaccine has been demonstrated to be effective and safe in several clinical studies [7, 8]. In a phase I/II trial involving 187 participants, Mittendorf EA [9] found that the disease-free survival (DFS) rate in the E75 vaccine group was different from that in the control group (89.7% vs. 80.2%). In addition, the GP2 peptide vaccine, known as p654 with a sequenced IISAVVGIL, was confirmed to induce patients with breast cancer to generate specific CD8^+^ T-cells [10, 11]. Mittendorf EA [12]verified the clinical efficacy of the GP2 peptide vaccine in a clinical study involving 180 patients, and DFS was 88% higher than that of the control group (80%). The AE37 peptide vaccine was obtained by adding II-key peptide (LRMK) with a length of four amino acids on the AE36 base (the sequence is GVGSPYVSRLLGICL). Compared with AE36 vaccines, the ability of the AE37 vaccine to bind to human major histocompatibility complex class II was enhanced 250 times [13]. Few clinical trials have been conducted with AE37 [14]. In a phase I trial, Holmes [15] showed that AE37 elicited a strong immune response, and its delayed-type hypersensitivity reaction (DTH) increased to 56 mm^2^ after injection. The above studies indicated that the HER2 vaccine has fairly broad prospects for the treatment of breast cancer. However, there have been few systematic evaluations and meta-analyses on the efficacy of HER2 vaccines. Therefore, this study intended to systematically evaluate the immunogenicity and clinical efficacy of the E75 and GP2 vaccines.

## Materials and methods

### Protocol and registration

A protocol was formulated for this study, and it was registered in PROSPERO (https://www.crd.york.ac.uk/PROSPERO/) with number CRD42020218012.

### Criteria for literature retrieval

**Fig. 1:**
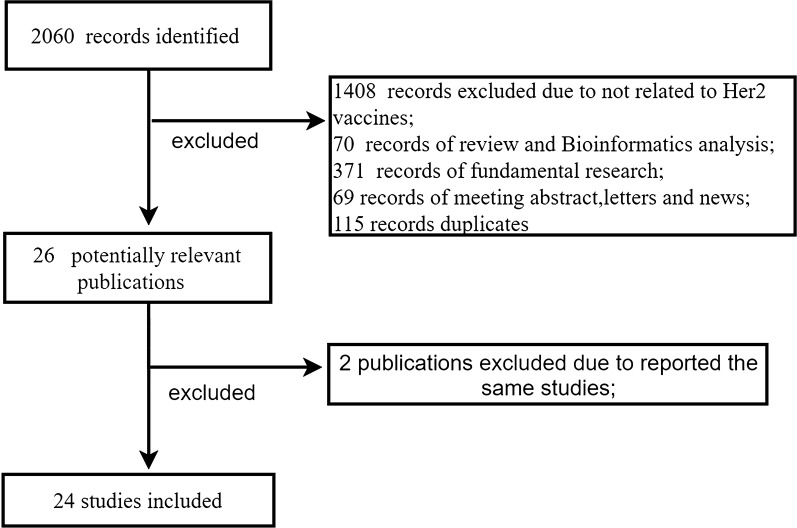
Flow diagram showing the procedure used to select trials

Search keywords included “breast cancer”, “breast neoplasia”, “Random”, “Randomized Trial”, “HER2”, “Erbb2”, and “vaccine”. Retrieval time was from the beginning of database construction until May 31, 2021. The retrieval language was English. The retrieval strategy was a combination of keywords and retrieval methods to improve recall and precision. Meeting summaries, reviews, case reports, letters, and unrelated studies were manually excluded. Relevant clinical research literature was searched in the Cochrane Library, PubMed, Medline, Embase, and Web of Science databases (Fig. 1 for details). The results were retrieved separately by two independent researchers and compared. If a dispute was raised, a third researcher was asked to resolve the discrepancy.

### Inclusion and exclusion criteria

#### Inclusion criteria

Clinical research literature published in journals starting on the date when the abovementioned database was established until May 31, 2021. The subjects of the study were female patients with breast cancer diagnosed by pathology without any other type of tumor. The literature used the following evaluation methods: changes in CD8 + T-cells before and after vaccine injection, measured value of the delayed-type hypersensitivity reaction (DTH), recurrence rate of tumor patients, overall survival rate, and disease-free survival rate (DFS) of patients.

#### Exclusion criteria

Reviews, meeting abstracts, basic studies and clinical studies unrelated to HER2 vaccine, and studies with unclear data description or poor quality. For literature reporting the same study multiple times, the latest publication was included for analysis.

### Clinical trial selection and data extraction

In this study, two independent researchers simultaneously searched the relevant literature, compared and evaluated the titles and abstracts, and conducted a full-text assessment for detailed analysis and data extraction of the studies that were suitable for inclusion criteria. When there were differences, the full texts were evaluated by a third researcher.

### Literature quality evaluation

The quality of the included studies was assessed according to the Cochrane risk bias assessment tool [16], which contained random sequence generation (selection bias), allocation concealment (selection bias), blinding of participants and personnel (performance bias), blinding of outcome assessment (detection bias), incomplete outcome data (attrition bias), selective reporting (reporting bias), and other biases.

### Statistical analysis

STATA15.1, RevMan 5.3 and Excel 2019 were used for meta-analysis of the original data obtained in the literature. The standard mean differences (SMDs) and corresponding 95% confidence intervals (CIs) were applied to calculate the immune response of the E75 and GP2 vaccines, and relative risk (RR) and 95% confidence interval (CI) were used to represent the results of the clinical efficacy analysis. Binary variables were analyzed using the Mantel–Haenszel method, and continuous variables were analyzed with the inverse variance method. Heterogeneity among studies was evaluated with the χ^2^-based *Q*-test and quantitative metric I^2^. Data with high heterogeneity (P < 0.10, I^2^ > 50%) were analyzed with a random effects model. Otherwise, a fixed effects model was used for analysis with test level = 0.05. The results of the meta-analysis were represented by forest plots, and publication bias was evaluated with Egger’s and Begg’s tests.

## Results

### Included studies

A total of 24 clinical studies were included in this study, all of which were phase I/II/III clinical studies on breast cancer. There were 1704 patients in the vaccinated group and 1248 patients in the control group, including patients with positive HER2 expression, negative HER2 expression, positive axillary lymph node(s), or negative axillary lymph node(s). Altogether, 1580 patients in the vaccinated group and 1156 patients in the control group were enrolled in 21 clinical trials of E75. Three clinical trials of the GP2 vaccine, including 124 patients in the vaccinated group and 91 patients in the control group, were enrolled (Table 1 for details). All enrolled patients had received standard therapy. Patients in the GP2-vaccinated group were injected with the GP2 vaccine + granulocyte–macrophage colony stimulating factor (GM-CSF), while patients in the control group were injected with GM-CSF only. Patients in the E75-vaccinated group were injected with the E75 vaccine + GM-CSF, and patients in the control group were injected with GM-CSF. The mean age of the patients was 41.8 yrs.

**Table 1:** Clinical information table of the enrolled study

	References	Phase	Sample size		Mean age	HLA		Lymph node		DTH (mm^2^)-case		DTH (mm^2^)-control		CD8^+^ T-cell (%)-case		Recurrence (%)		Overall survival (%)		DFS (%)	
			Case	Control		Case	Control	Case	Control	Pre	Post	Pre	Post	Pre	Post	Case	Control	Case	Control	Case	Control
GP2	^a^Mittendorf [12]/Tommy [10]	II	89	91	49	A2^+^	A2^+^	51N^+^	60N^+^	4.1	15.3	3.9	8	0.8	1.5	5.12	2.35	92.13	95.6	82.9	80.4
	Clifton [30]	I	17	0	47	A2^+^, A3^+^		13N^+^		28	68			46^*^10^6^	144^*^10^6^						
	Carmichael [11]	I	18	0	47	A2^+^		18N^−^		0	27.5			0.4	1.1						
GP2 Total	3 trials		124	91																	
E75	Clifton [7]	II	136	139	51	A2^+^, A3^+^, A24^+^, A26^+^		81N^+^	88N^+^	1.6	7.9	2	2.1	0.025	0.09	8.82	14.4			89.8	83.8
	Clifton [32]	II	81	69	52	A2^+^, A3^+^, A24^+^, A26^+^	A2^+^, A3^+^, A24^+^, A26^+^	49N^+^	44N^+^												
	Mittendorf [8]	III	382	376	51.8	A2^+^, A3^+^	A2^+^, A3^+^	373N^+^	366N^+^							6.3	9.8			77.1	77.5
	Mittendorf [9]	I, II	108	79	55	A2^+^, A3^+^	A2^−^, A3^−^	53N^+^	44N^+^	1.2	13.7	0.4	1.7			10	20			89.7	80.2
	Mittendorf [33]	I, II	106	76	55	A2^+^, A3^+^	A2^−^, A3^−^	51N^+^	43N^+^							5.6	13.1			94.3	86.8
	Holmes [34]	I, II	53	0	58	A2^+^, A3^+^		27N^+^		NA	99.4			0.3	0.99						
	Patil [22]	I, II	106	81	56	A2^+^, A3^+^	A2^−^, A3^−^	46N^+^	53N^+^	3	14	3.2	3.5			8.3	14.8				
	Gates [35]	I	24	22	63	A2^+^	A2^−^			5	31.5	1.6	9.7			28.6	26.3	95.2	100		
	Benavides [36]	II	85	66	54	A2^+^, A3^+^	A2^−^,	49N^+^	42N^+^	4	14.84	NA	1.1	0.73	1.83	11.7	18.2	98.8	92.42		
	Peoples [20]	II	101	85	57	A2^+^, A3^+^	A2^−^, A3^−^	45N^+^	46N^+^	0.5	14	1.4	2.1	0.39	1.8	5.6	14.2	99	95.1	92.5	77
	Holmes [23]	II	99	0		A2^+^, A3^+^		48N^+^		2.7	21.5			0.416	1.78	10.1					
	Amin [27]	II	101	85		A2^+^, A3^+^	A2^−^, A3^−^	45N^+^	46N^+^	12.8	13.625	NA	2.64	0.42	1.79	8.3	14.8	99	93.8	88	58
	Stojadinovic [37]	I	16	NA	52	A2^+^		16N^+^		NA	22.3	NA	3	0.36	0.65						
	Dehqanzada [38]	I, II	36	0	59.7	A2^+^	A2^−^	12N^+^													
	Dehqanzada [39]	I, II	32	0	57	A2^+^		10N^+^													
	Hueman [40]	II	7	17		A2^+^	A2^−^	7N^−^	17N^−^					58.8	67.4						
	Hueman [41]	I	22	22		A2^+^	A2^−^	22N^−^	22N^−^					0.75	1.2						
	Mittendorf [42]	I	44	0		A2^+^	A2^−^	24N^+^						0.5	1.68						
	Peoples [21]	I	24	29		A2^+^	A2^−^	24N^+^	29N^+^	NA	33	NA	7	0.56	1.66	8.3	20.7	100	93	85.7	59.8
	Murray [43]	I	13	0	48	A2^+^		6N^+^		NA	7.83										
	Knutson [44]	I	4	10	48	A2^+^	A2^+^														
E75 Total	21 trials		1580	1156																	

*NA* not available, *DTH* delayed hypersensitivity reaction, *HLA* human leukocyte antigen, *DTH* delayed-type hypersensitivity reaction, *DFS* disease-free survival rate, *HLA-A2*^*±*^ A2^±^ human leukocyte antigen A2 positive/negative, *HLA-A3*^*±*^ A3^±^ human leukocyte antigen A3 positive/negative , *HLA-A24*^*±*^ A24^±^ human leukocyte antigen A24 positive/negative, *HLA-A26*^*±*^ A26^±^ human leukocyte antigen A26 positive/negative

^a^Tommy A(2020) and Mittendorf EA(2016) ^17^reported the same clinical trial

### Immune responses

**Fig. 2:**
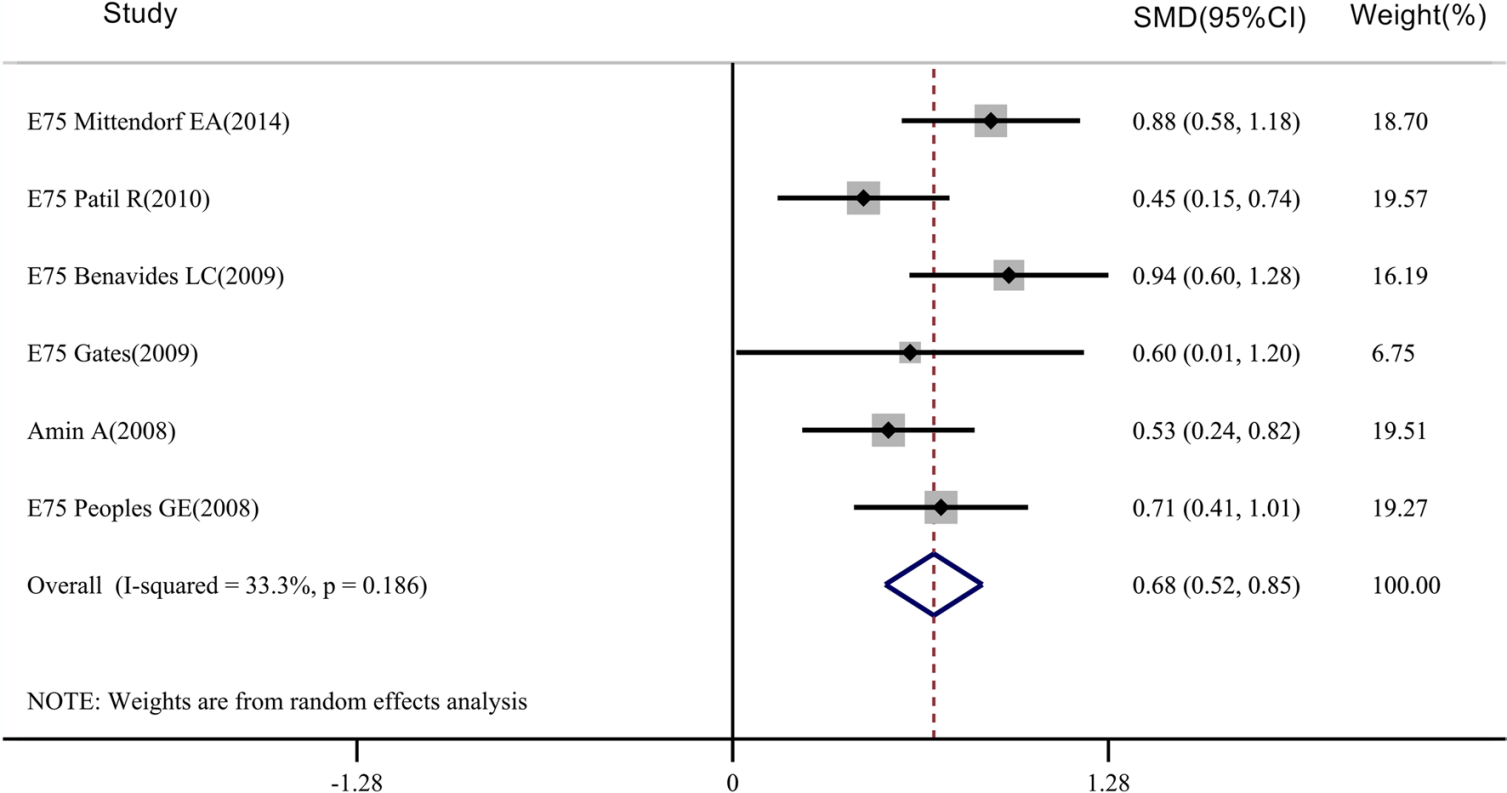
Meta-analysis of the E75 vaccine delayed hypersensitivity reaction (DTH): STATA 15.0 was used to analyze the 6 clinical studies. The relative standard mean difference (SMD) value and the 95% confidence interval (CI) were used to describe the effect of the vaccine on vaccinated and control patients. Heterogeneity was tested with the Q measurement method (P = 0.186) and I^2^ measurement method (I^2^ = 33.3%). Meta-analysis of DTH of 685 patients in the vaccinated group and 587 patients in the control group showed that the DTH of the two groups was different (*P* < 0.05)

**Fig. 3:**
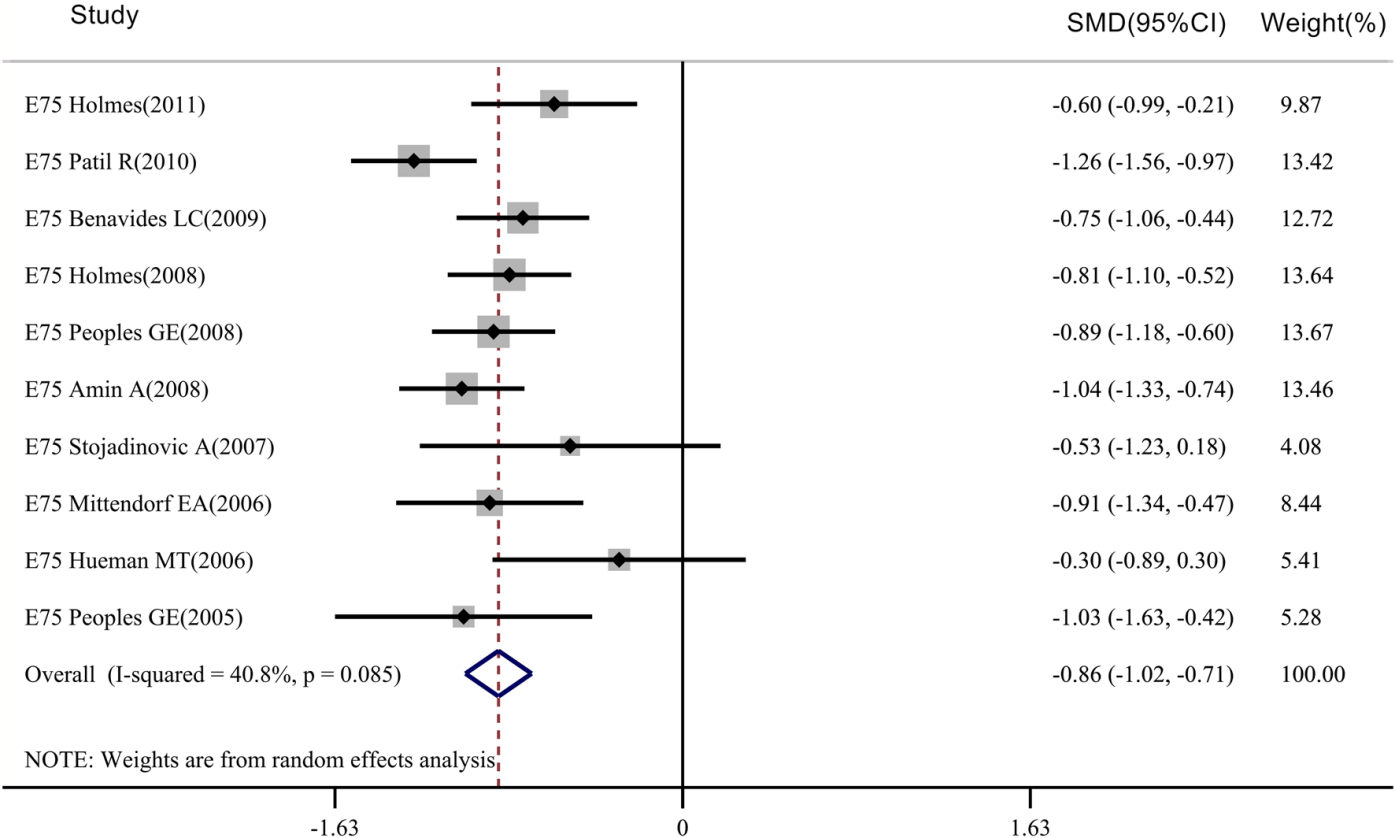
Meta-analysis of CD8 + T-cell levels after injection of the E75 vaccine: STATA 15.0 was used to analyze the 10 clinical studies. The relative standard mean difference (SMD) value and the 95% confidence interval (CI) were used to describe the effect of the vaccine on vaccinated and control patients. Heterogeneity was tested using the Q measurement method (P = 0.085) and the I^2^ measurement method (I^2^ = 40.8%). Meta-analysis of CD8^+^ T-cell levels in 651 patients in the vaccinated group found that there were differences in CD8^+^ T-cell levels before and after injection (*P* < 0.05)

**Fig. 4:**
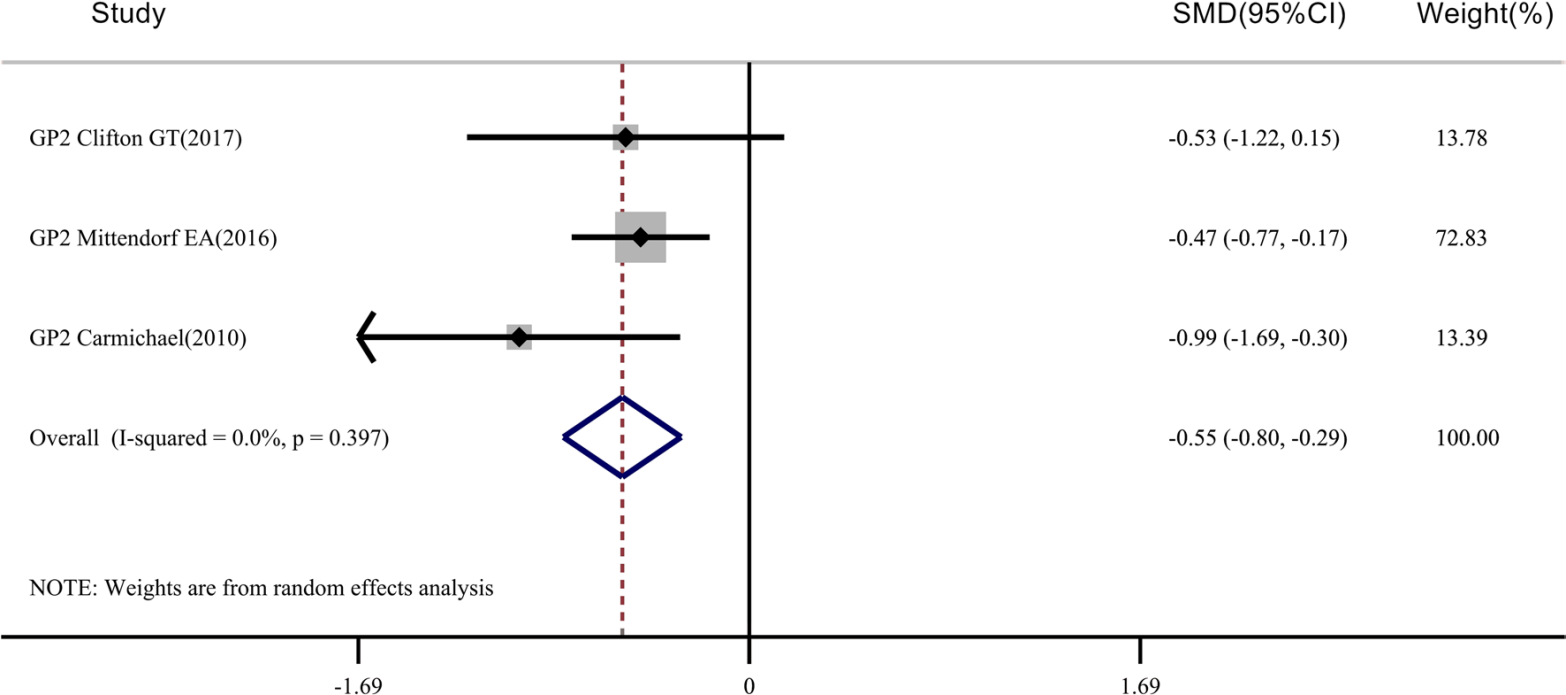
Meta-analysis of CD8 + T-cell levels after injection of the GP2 vaccine: STATA 15.0 was used to analyze the 3 clinical studies. The relative standard mean difference (SMD) value and the 95% confidence interval (CI) were used to describe the effect of the vaccine on vaccinated and control patients. Heterogeneity was tested using the Q measurement method (P = 0.397) and the I^2^ measurement method (I^2^ = 0.0%). A meta-analysis of CD8^+^ T-cell levels of 124 patients in the vaccinated group found that there were differences in CD8^+^ T-cell levels before and after injection (*P* < 0.05)

The immune responses of HER2 vaccines are mainly evaluated by delayed-type hypersensitivity reaction (DTH) and changes in CD8^+^ T-cell numbers [17, 18]. A meta-analysis was conducted on the DTH of 685 patients in the vaccinated group and 587 patients in the control group of the E75 vaccine (Fig. 2). The results showed that the DTH of the E75 vaccine in the vaccinated group was higher than that in the control group (SMD = 0.685 95% CI 0.52–0.85, P_Heterogeneity_ = 0.186, P_DTH_ < 0.05). Changes in CD8^+^ T-cell numbers before and after injection can reflect the strength of the immune response to therapeutic vaccines [19]. A comparison of the E75 vaccine in 651 patients in the vaccinated group before and after the injection of the vaccine was performed (Fig. 3), and there was a significant difference in the change in CD8^+^ T-cell numbers before and after injection (SMD = − 0.864, 95% CI − 1.02 to − 0.709, P_Heterogeneity_ = 0.085, P_CD8+ T cell_ < 0.05). In addition, the number of CD8^+^ T-cells in patients receiving the GP2 vaccine also showed a significant difference before and after injection. (SMD =− 0.584, 95% CI − 0.803 to − 0.294, P_Heterogeneity_ = 0.397, P_CD8+ T cell_ < 0.05) (Fig. 4). The results showed that E75 and GP2 vaccines both had strong immunogenicity.

### Clinical outcomes

**Fig. 5:**
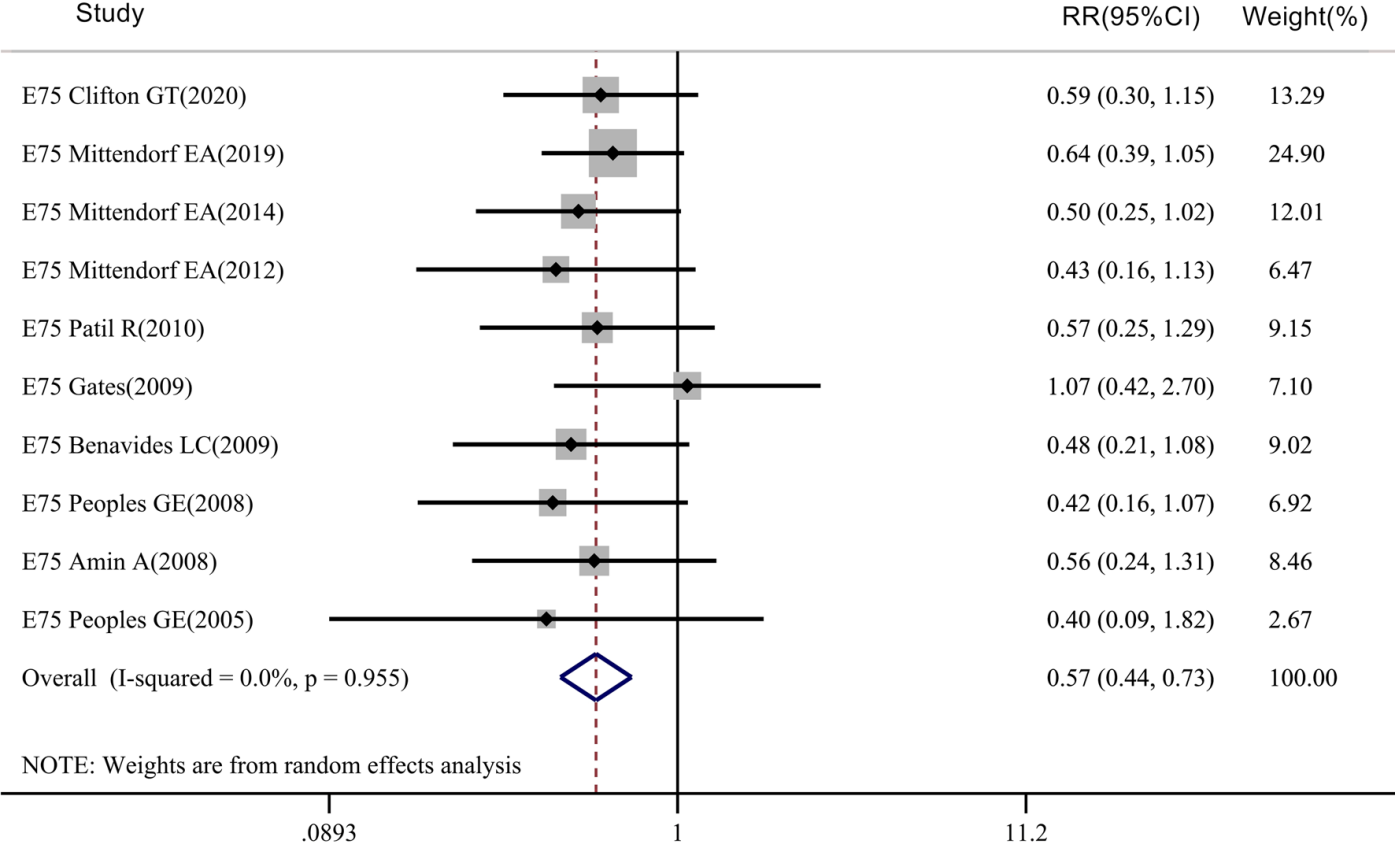
Meta-analysis of recurrence rate of patients with E75 vaccine: STATA 15.0 was used to analyze the 10 clinical studies. The relative risk (RR) value and the 95% confidence interval (CI) were used to describe the effect of the vaccine on vaccinated and control patients. Heterogeneity was tested using the Q measurement method (P = 0.955) and the I^2^ measurement method (I^2^ = 0.0%). A meta-analysis of the recurrence rate of 1517 patients in the vaccinated group and 1217 patients in the control group showed that the recurrence rate was different between the two groups (*P* < 0.05)

The meta-analysis of the E75 vaccine recurrence rate included 10 clinical trials, with follow-up times ranging from 17 to 60 months, including 1517 patients in the vaccinated group and 1217 patients in the control group (Fig. 5). The results showed that the recurrence rate in the E75-vaccinated group was different from that of the control group (RR = 0.568, 95% CI 0.444–0.727, P_Heterogeneity_ = 0.955, P_recurrence_ < 0.05).

**Fig. 6:**
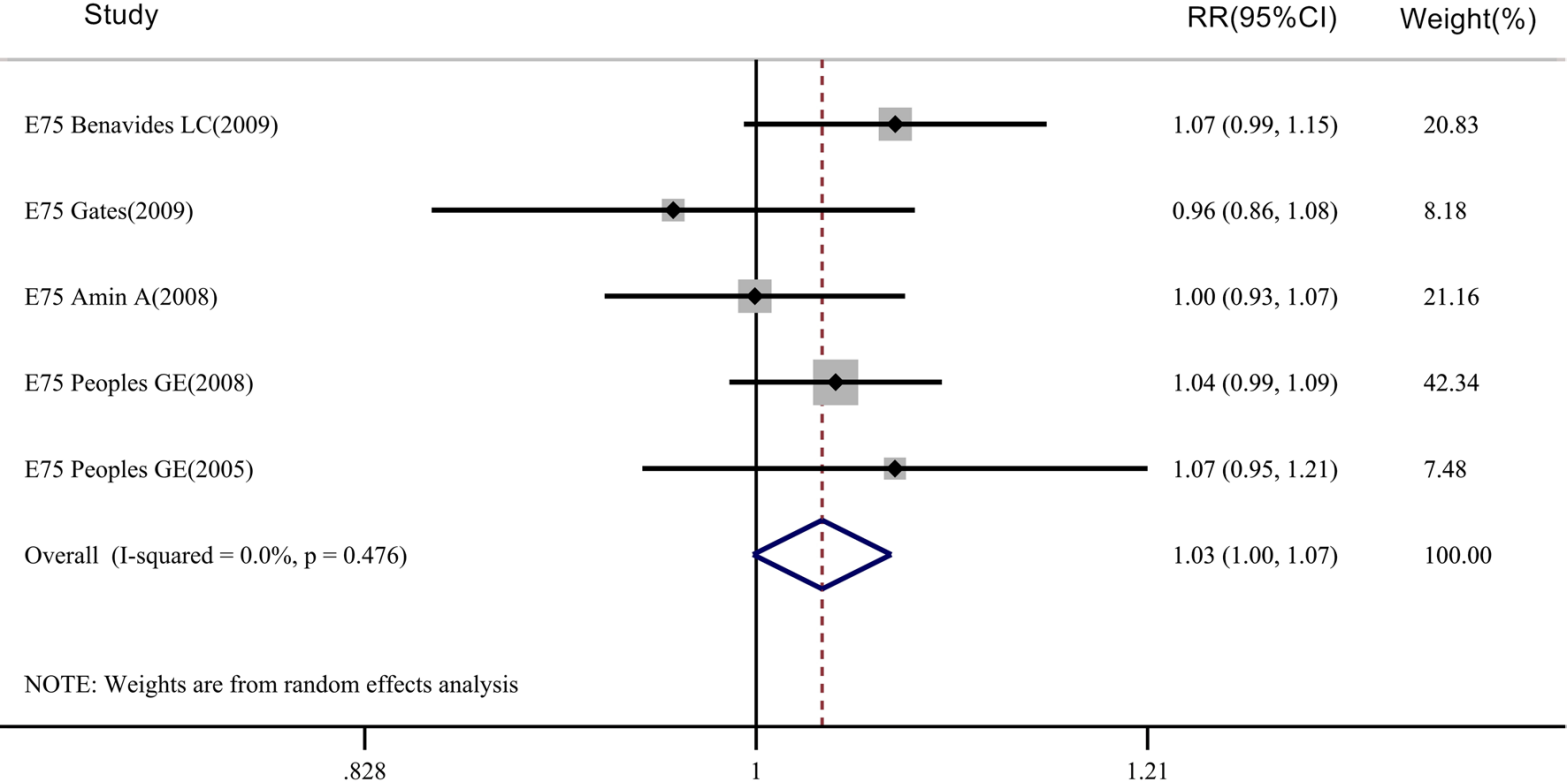
Meta-analysis of overall survival rate after E75 vaccine injection: STATA 15.0 was used to analyze the five clinical studies. The relative risk (RR) value and the 95% confidence interval (CI) were used to describe the effect of the vaccine on vaccinated and control patients. Heterogeneity was tested using the Q measurement method (P = 0.476) and the I^2^ measurement method (I^2^ = 0.0%). A meta-analysis of the overall survival rate of 335 patients in the vaccinated group and 287 patients in the control group showed that the overall survival rate was not different between the two groups (*P* > 0.05)

**Fig. 7:**
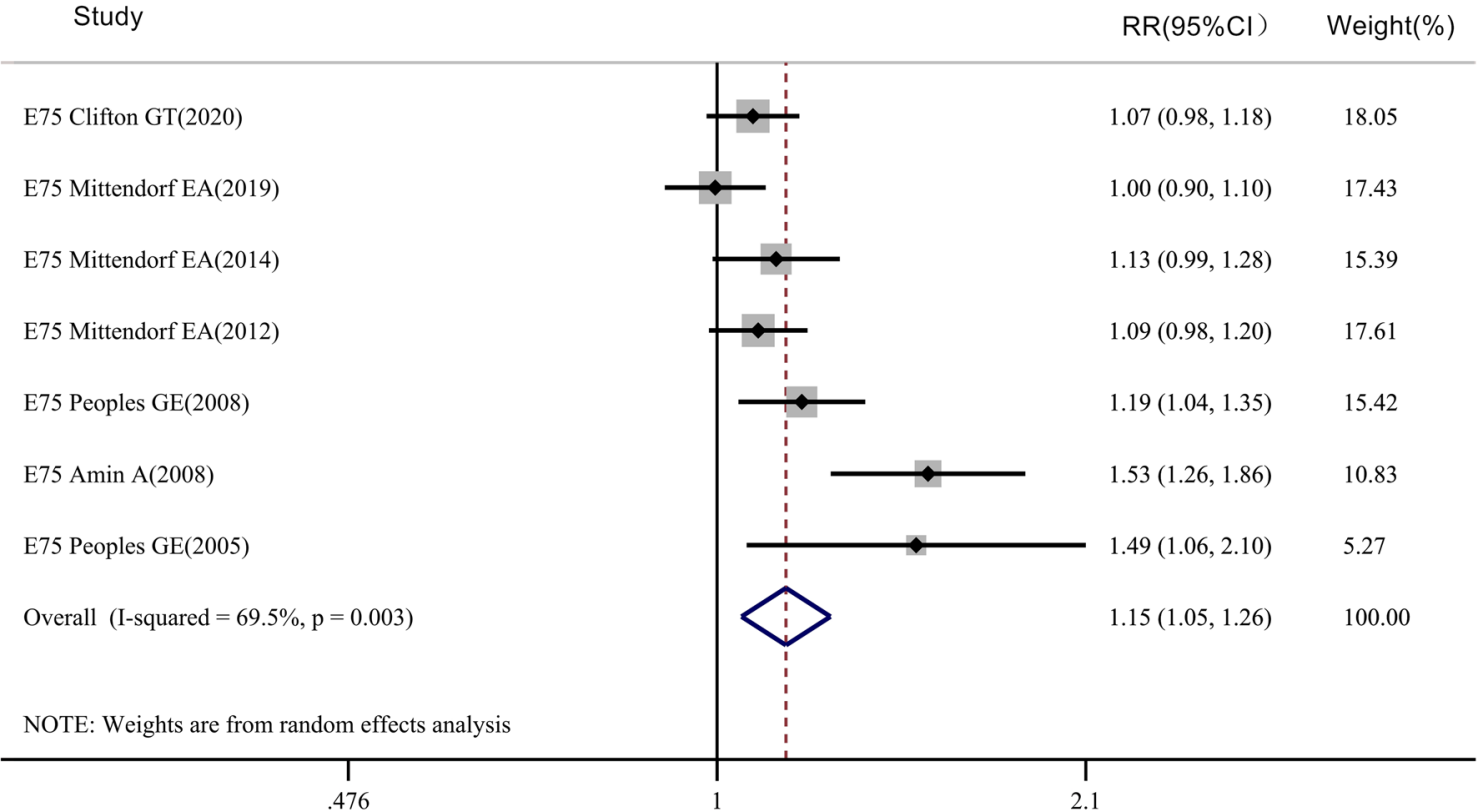
Meta-analysis of disease-free survival (DFS) after E75 vaccine injection: STATA 15.0 was used to analyze the nine clinical studies. The relative risk (RR) value and the 95% confidence interval (CI) were used to describe the effect of the vaccine on vaccinated and control patients. Heterogeneity was tested by the Q measurement (P = 0.003) and the I^2^ measurement (I^2^ = 69.5%). A meta-analysis of the disease-free survival of 962 patients in the vaccinated group and 852 patients in the control group showed that the disease-free survival rate was different between the two groups (*P* < 0.05)

The meta-analysis of overall survival (OS) rate included 5 clinical studies on the E75 peptide vaccine (Fig. 6), with 335 patients in the vaccinated group and 287 patients in the control group. However, we found that for the E75 vaccine (RR = 1.032, 95% CI 0.998–1.067, P_Heterogeneity_ = 0.476, P_OS_ > 0.05), there was no significant difference between the two groups in terms of overall survival rate. For the DFS rate, 7 clinical trials were included in the meta-analysis of disease-free survival (DFS), including 962 patients in the vaccinated group and 852 patients in the control group (Fig. 7). There was a significant difference between the two groups for the DFS rate of the E75 vaccine (RR = 1.149, 95% CI 1.050–1.256, P_Heterogeneity_ = 0.003, P_DFS_ < 0.05).

### Side effects

A total of 12 studies reported local and systemic toxicities of tumor vaccines (three studies for GP2 vaccines and 9 for E75 vaccines). Grade 1 local and systemic toxic reactions were the most common toxic side effects, and grade 2 toxic reactions were also found in most studies. Eight trials reported grade 3 systemic toxicity, including Mittendorf EA et al. [12], 1%, Tommy et al. [10], 1%, Peoples GE et al. [20], 2%, Peoples GE et al. [21], 1.8%, Patil R et al. [22], 2.3%, Mittendorf EA et al. [8], 5.9%, Mittendorf EA et al. [9], 1%, and Holmes et al. [23], 1%. One clinical trial reported grade 4 toxicity. (Mittendorf EA et al. [8], 0.3%). The above data indicated that the GP2 and E75 vaccines had low toxicity, which provided a basis for clinical application.

### Study quality

Egger’s test (t = 0.99, P = 0.350) and Begg’s test (Z = 1.79, P = 0.074) indicated that no publication bias existed in the studies included. The combined results of DTH (I^2^ = 33.3%, P = 0.186) and change in CD8^+^ T-cell number (I^2^ = 40.8%, P = 0.085) in the E75 vaccine had mild heterogeneity. The combined result of DFS rate in the E75 vaccine had moderate heterogeneity (I^2^ = 69.5%, P = 0.003).

## Discussion

With the wide application of targeted therapy, the problem of drug resistance has become increasingly significant [24]. Investigation and clinical application of the HER-2 vaccine can provide a new direction of treatment for patients with breast cancer. The efficacy of vaccines was evaluated by immunogenicity and clinical outcome [8]. Delayed hypersensitivity reaction refers to the redness, sclerosis, and even blisters and necrosis at the injection site 48–72 h after injection. This was a local hypersensitive inflammatory response caused by the binding of sensitized T-lymphocytes to antibodies [25]. This was one of the most common indices to evaluate the immunogenicity of therapeutic vaccines. Multiple clinical trials have shown that the E75 vaccine can elicit an immune response in the treatment of breast cancer [26]. In a phase II clinical trial of the E75 vaccine containing 196 patients, Amin A [27] found that the redness of the vaccinated nonrecurrent group increased to 13.5 ± 1.5 mm^2^, and another study found that DTH responses ≥ 10 mm^2^ were related to vaccine immunity [28]. However, in another phase II clinical trial involving 275 patients with breast cancer, Clifton GT [7] found that the mean DTH response in the E75 vaccine group was 7.9 mm^2^. To confirm its immunogenicity, we conducted a meta-analysis on DTH of the E75 vaccine and found that there were differences between the vaccinated group and the control group in DTH (SMD = 0.685 95% CI 0.52–0.85, P_Heterogeneity_ = 0.186, P_DTH_ < 0.05), which showed that the E75 vaccine could elicit a DTH response in six included studies. Changes in CD8^+^ T-cells reflect the strength of the immune response after vaccination [29]. In this study, we found that CD8^+^ T-cell numbers were different before and after injection in ten studies (SMD = − 0.864, 95% CI − 1.02 to − 0.709, P_Heterogeneity_ = 0.085, P_CD8+ T cell_ < 0.05).

Regarding clinical outcomes, in a phase I/II clinical trial of 187 patients with breast cancer, Patil R [22] found that the E75 vaccine group was associated with an 8.3% recurrence rate compared with 14.8% in the control group, and the overall survival rate was 99% in the vaccinated group compared with 93.8% in the control group. However, its long-term efficacy in patients with breast cancer is still controversial. Mittendorf EA [8], in a multicenter clinical trial including 758 participants, concluded that there was no significant difference in DFS rate between a E75 vaccinated group and a control group. In view of this, a meta-analysis of clinical outcomes including recurrence rate, OS rate, and DFS rate of the E75 vaccine was conducted, and it found that the E75 vaccine not only differed between vaccinated group and control group in recurrence rate in ten studies (RR = 0.568, 95% CI 0.444–0.727, P_Heterogeneity_ = 0.955, P_recurrence_ < 0.05) but also in DFS rate in seven studies (RR = 1.149, 95% CI 1.050–1.256, P_Heterogeneity_ = 0.003,P_DFS_ < 0.05). However, the overall survival rate was not different between the two groups. (RR = 1.032, 95% CI 0.998–1.067, P_Heterogeneity_ = 0.476, P_OS_ > 0.05). The results above confirmed the immunogenicity and clinical efficacy of the E75 vaccine in patients with breast cancer.

In terms of the GP2 vaccine, Clifton GT [30] found that it could elicit an immune response in patients with breast cancer in a phase I clinical trial, thus demonstrating its immunogenicity. However, studies show that the ability of the GP2 vaccine to induce the generation of specific antitumor cells was relatively weaker than that of the E75 and AE37 vaccines [12]. Therefore, we conducted a meta-analysis of the GP2 vaccine and found that the GP2 vaccine could elicit a strong immune response resulting in a change in CD8^+^ T-cell numbers in three studies before and after injection (SMD = − 0.584, 95% CI − 0.803 to − 0.294, P_Heterogeneity_ = 0.397, P_CD8+ T cell_ < 0.05). Because of the limited number of clinical trials on the GP2 vaccine, data for further analysis of the clinical efficacy of the GP2 vaccine are insufficient. Mittendorf EA [12] verified the clinical efficacy of the GP2 peptide vaccine in a clinical trial involving 180 patients, and DFS was 88% higher than that of the control group (80%). However, with long-term follow-up, Tommy A [10] refuted the conclusion that the DFS of the vaccinated and control groups was not significantly different. The clinical efficacy of the GP2 vaccine needs to be confirmed with more clinical trials.

According to the Common Terminology Criteria for Adverse Events (Version 5.0), Grade 3 indicates a severe or medically significant disabling event, limiting self-care, but not immediately life-threatening, with hospitalization or prolongation of hospitalization indicated; Grade 4 indicates life-threatening consequences and urgent intervention indicated [31]. We found that the study that reported the most participants of grade 3 was the one by Mittendorf EA et al. [8], with 5.9%, and the only study that reported grade 4 was the one by Mittendorf EA et al. [8], with 0.3%. The results showed that the E75 and GP2 vaccines had low local and systemic toxicities and were safe for patients with breast cancer.

In the mechanism of action of peptide vaccines, the binding of APCs and T-helper cells requires mutual recognition of antigens and human leukocyte antigen (HLA) molecules [3]. The type of human HLA must be considered in the application of peptide vaccines. The E75 and GP2 vaccines were only available to people with HLA-A2^+^ and HLA-A3^+^. This was one of the limitations of the widespread application of peptide vaccines. Pre-existing immunity to the vaccine referred to peptide-specific dimer levels ≥ 0.3% before vaccination. According to Carmichael [11], pre-existing immunity decreases the ability of patients to generate specific CTLs after vaccination, which may reduce the efficacy of vaccines. In addition, drug resistance, toxicity and high costs are factors that need to be considered.

The limitations of this study were related to the quantity and quality of studies included about GP2 vaccines. The conclusion of the GP2 vaccine might be less accurate for the relatively small number of studies included. The effect of GP2 vaccines on long-term treatment needs to be evaluated and analyzed with more clinical trials. Although the disease-free survival rate with the E75 vaccine had moderate heterogeneity, these studies are supportive of the efficacy of the E75 vaccine. The heterogeneity among studies possibly comes from the stage of breast cancer, age, dose of the vaccine, clinical nodal status, and pre-existing immune status to the vaccine.

## Conclusion

Our meta-analysis results demonstrated that the E75 vaccine was effective and safe in patients with breast cancer. The GP2 vaccine could elicit a strong immune response, but its clinical efficacy needs to be confirmed with more clinical trials.

## Data Availability

All data analyzed in the study are available in the published manuscripts.
